# Correction: Ho et al. NIR-Triggered Generation of Reactive Oxygen Species and Photodynamic Therapy Based on Mesoporous Silica-Coated LiYF_4_ Upconverting Nanoparticles. *Int. J. Mol. Sci.* 2022, *23*, 8757

**DOI:** 10.3390/ijms27031219

**Published:** 2026-01-26

**Authors:** Tsung-Han Ho, Chien-Hsin Yang, Zheng-En Jiang, Hung-Yin Lin, Yih-Fung Chen, Tzong-Liu Wang

**Affiliations:** 1 Department of Chemical and Materials Engineering, National Kaohsiung University of Science and Technology, Kaohsiung 807, Taiwan; thho@nkust.edu.tw; 2 Department of Chemical and Materials Engineering, National University of Kaohsiung, Kaohsiung 811, Taiwan; yangch@nuk.edu.tw (C.-H.Y.); m1105619@mail.nuk.edu.tw (Z.-E.J.); linhy@nuk.edu.tw (H.-Y.L.); 3 Graduate Institute of Natural Products, Kaohsiung Medical University, Kaohsiung 807, Taiwan; yihfungchen@kmu.edu.tw

## Error in Figure

In the original publication [1], there was a mistake in Figure 1 as published. The Authors’ XRD patterns presented in Figure 1 of the IJMS article were directly derived from the graduate student’s research results. However, after rechecking the XRD raw data of UCNP@mSiO_2_, the authors discovered that he mistakenly used the XRD patterns of ligand-free-type UCNPs instead of those for UCNP@mSiO_2_. The mistake of misplacing previous data in his research results may be attributed to the use of smooth and anti-aliasing functions in the Origin Graphs by this student. It is believed that the similarity between the XRD patterns of UCNP@mSiO_2_ and ligand-free UCNPs becomes apparent after applying smooth and anti-aliasing functions, which might have led to his mistake. The corrected Figure 1 appears below.

## Missing Citation

In the original publication, Figure 6a,e were not cited. The citation has now been inserted into Section 3.4, Paragraph 4 and should read as follows:

For completeness of the comparison, two figures from the authors’ previous work [50] are included as shown in Figure 6a,e.

**Acknowledgments:** The authors gratefully acknowledge the kind help of Shuo-Yang Hong in some parts of this paper.

H.-Y. Lin confirms that his contributions to the manuscript remain unchanged following the correction. The authors state that the scientific conclusions are unaffected. This correction was approved by the Academic Editor. The original publication has also been updated.

## Figures and Tables

**Figure 1:**
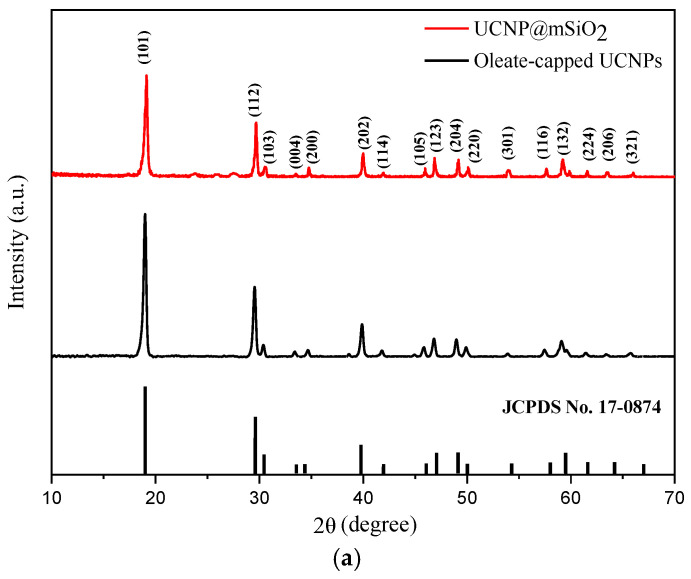

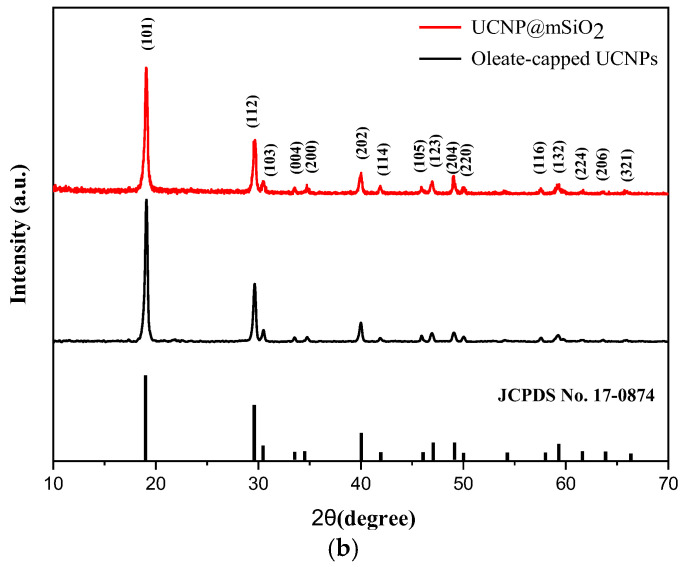
Wide-angle X-ray diffractograms of the oleate-capped and mSiO_2_-coated core/shell nanoparticles. (**a**) LiYF_4_:Yb^3+^_0.25_,Ho^3+^_0.01_@LiYF_4_:Yb^3+^_0.2_, (**b**) LiYF_4_:Yb^3+^_0.25_,Er^3+^_0.01_@LiYF_4_:Yb^3+^_0.2_.
