# Supplementary material for: NIR-Triggered Generation of Reactive Oxygen Species and Photodynamic Therapy Based on Mesoporous Silica-Coated LiYF4 Upconverting Nanoparticles
Source: Int J Mol Sci. 2022 Aug 6;23(15):8757. doi: 10.3390/ijms23158757 (PMC9368848; doi:10.3390/ijms23158757)
Supplement: Supplementary file 1 [file ijms-23-08757-s001.zip › ijms-1848714-supplementary.pdf]

Supporting Information

NIR-Triggered Generation of Reactive Oxygen Species and Photodynamic Therapy Based on Mesoporous Silica-Coated $LiYF_4$ Upconverting Nanoparticles

Tsung-Han Ho, Chien-Hsin Yang, Zheng-En Jiang, Hung-Yin Lin, Yih-Fung Chen, Tzong-Liu Wang*

(a)

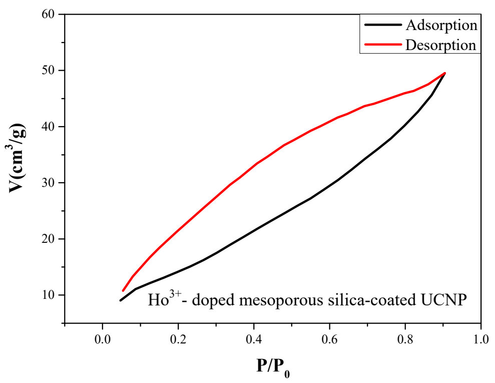

(b)

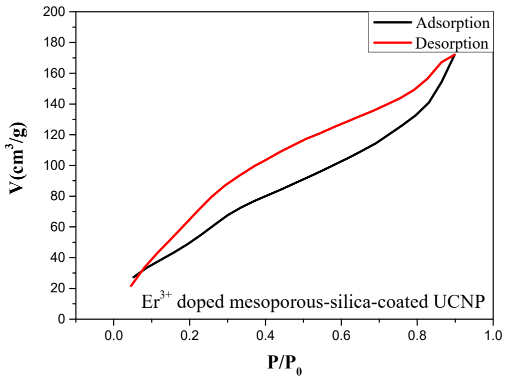

(c)

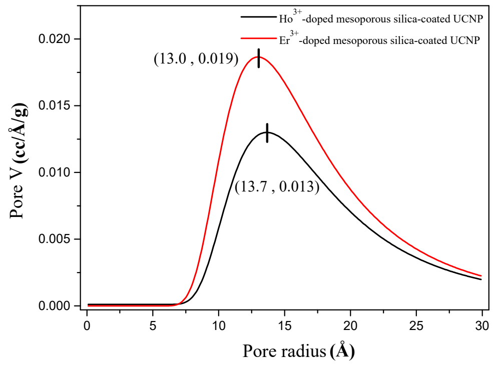

Figure S1. N_2 adsorption/desorption isotherm of (a) Ho^{3+}-doped UCNP@mSiO_2 and (b) Er^{3+}-doped UCNP@mSiO_2. (c) BJH pore size analysis for Ho^{3+}-doped UCNP@mSiO_2 and Er^{3+}-doped UCNP@mSiO_2.

(a)

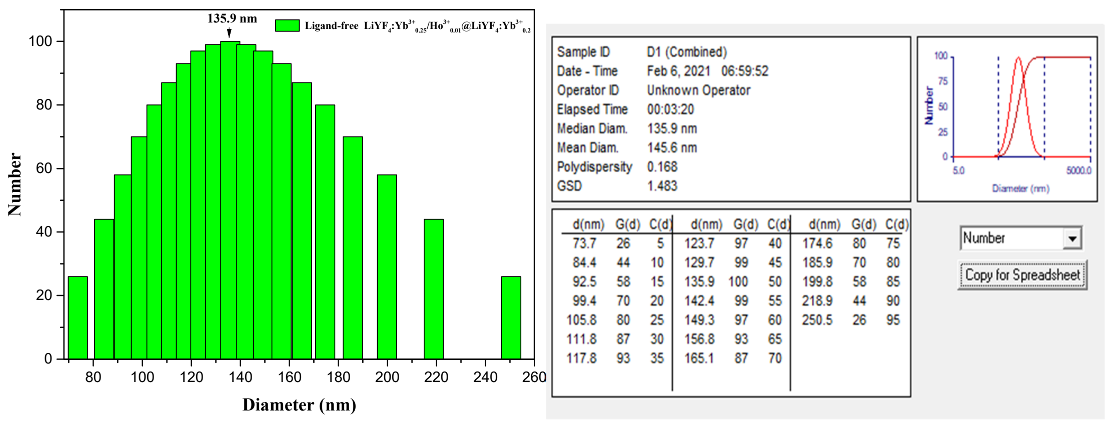

(b)

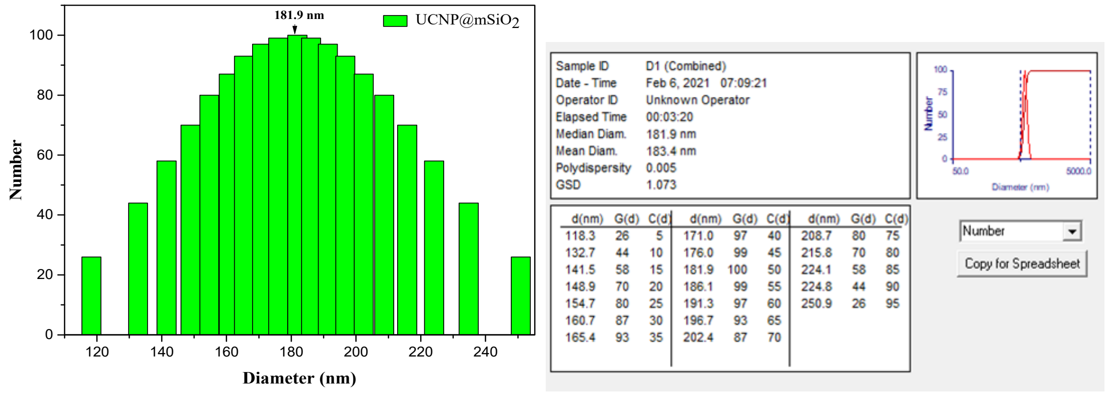

Figure S2. DLS analysis of $LiYF_4:Yb^{3+}{}_{0.25},Ho^{3+}{}_{0.01}@LiYF_4:Yb^{3+}{}_{0.2}$ nanoparticles. (a) Ligand-free core/shell UCNPs. (b) $mSiO_2$-coated core/shell nanoparticles.

(a)

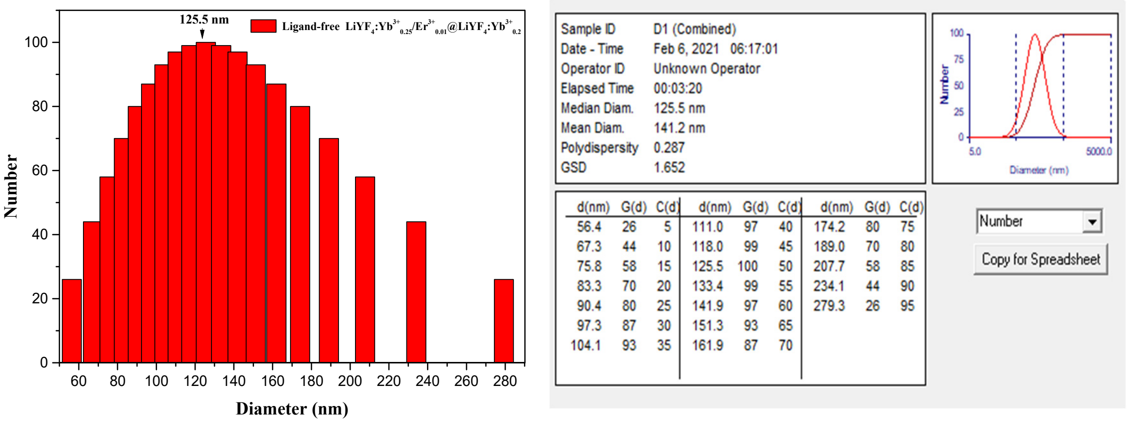

(b)

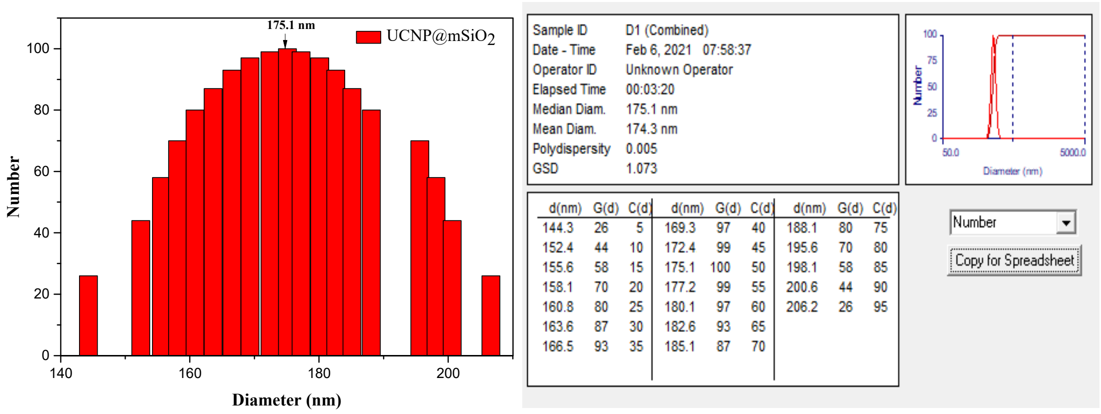

Figure S3. DLS analysis of $LiYF_4$:$Yb^{3+}_{0.25}$,$Er^{3+}_{0.01}$@$LiYF_4$:$Yb^{3+}_{0.2}$ nanoparticles. (a) Ligand-free core/shell UCNPs. (b) $mSiO_2$-coated core/shell nanoparticles.

(a)

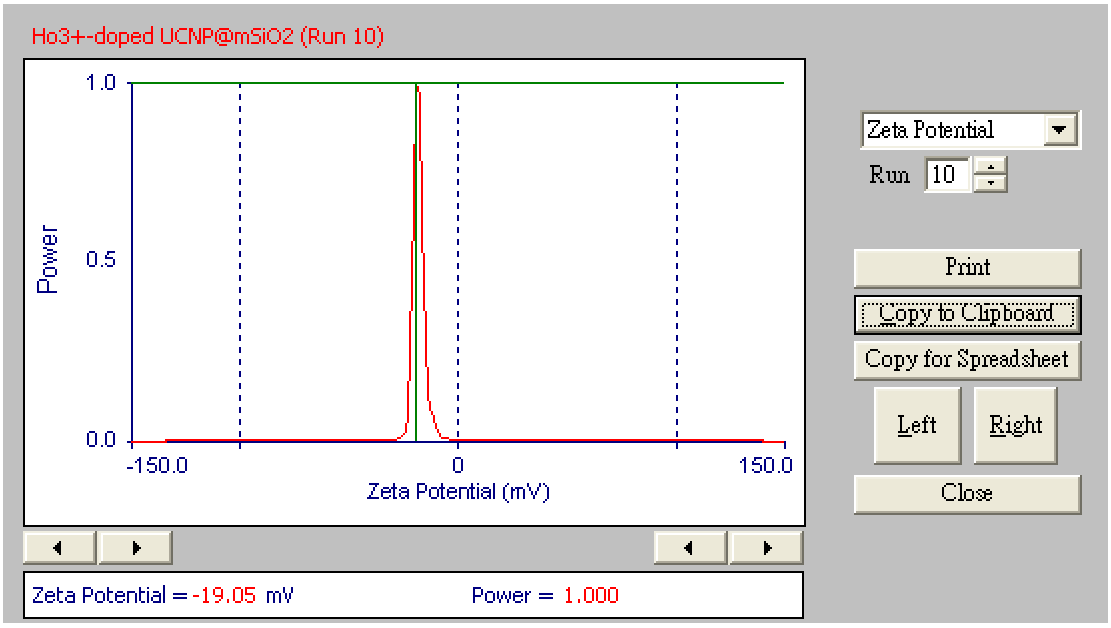

(b)

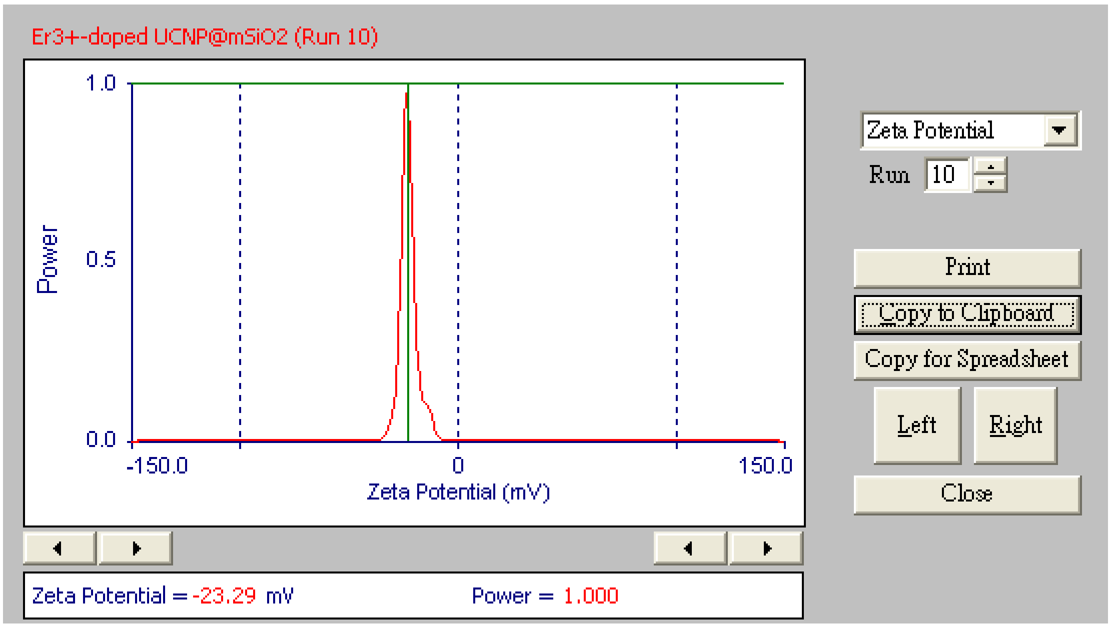

Figure S4. Zeta potential measurements of (a) $mSiO_2$-coated $LiYF_4$: $Yb^{3+}_{0.25}/Ho^{3+}_{0.01}$ @ $LiYF_4$:$Yb^{3+}_{0.2}$ and (b) $mSiO_2$-coated $LiYF_4$: $Yb^{3+}_{0.25}/Er^{3+}_{0.01}$ @ $LiYF_4$:$Yb^{3+}_{0.2}$ UCNPs.

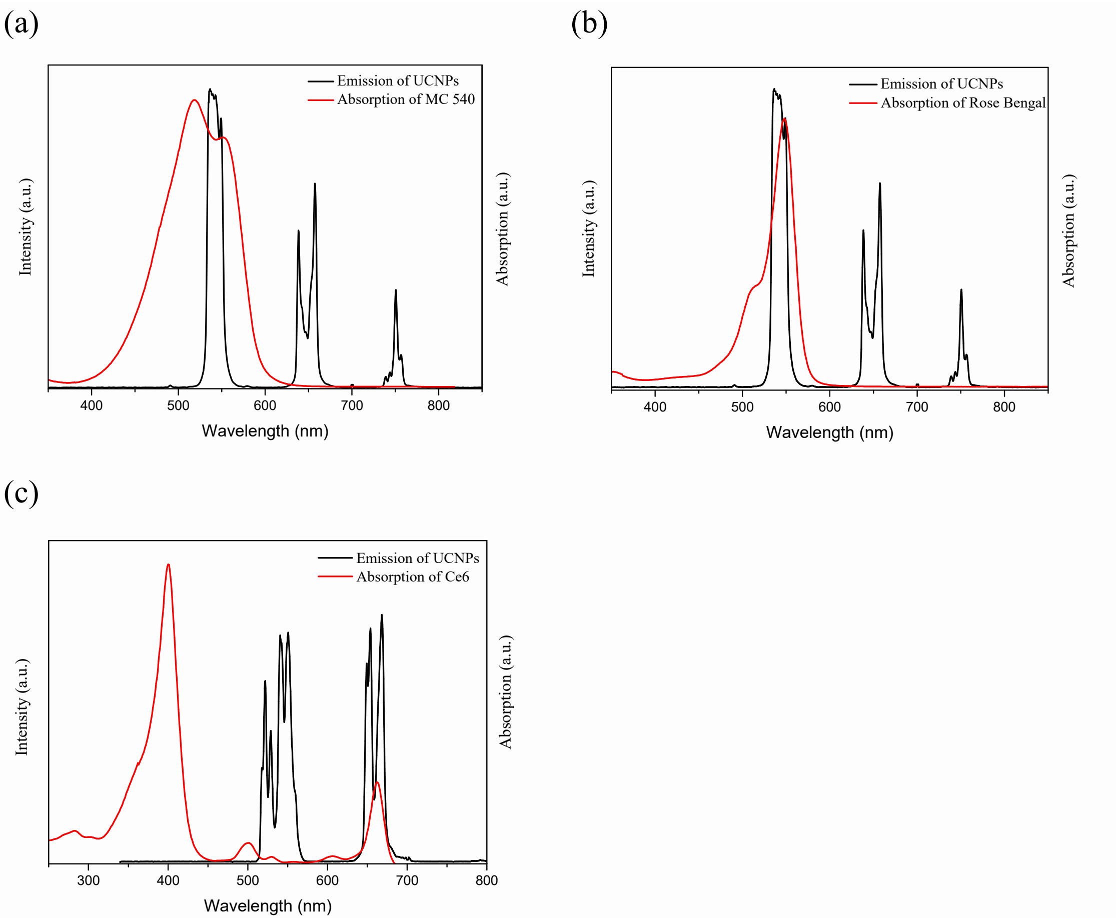

Figure S5. (a) The absorption spectrum of MC 540 and the emission spectrum of the Yb^{3+}/Ho^{3+}-doped UCNPs in water. (b) The absorption spectrum of rose bengal and the emission spectrum of the Yb^{3+}/Ho^{3+}-doped UCNPs in water. (c) The absorption spectrum of Ce6 and the emission spectrum of the Yb^{3+}/Er^{3+}-doped UCNPs in ethanol.

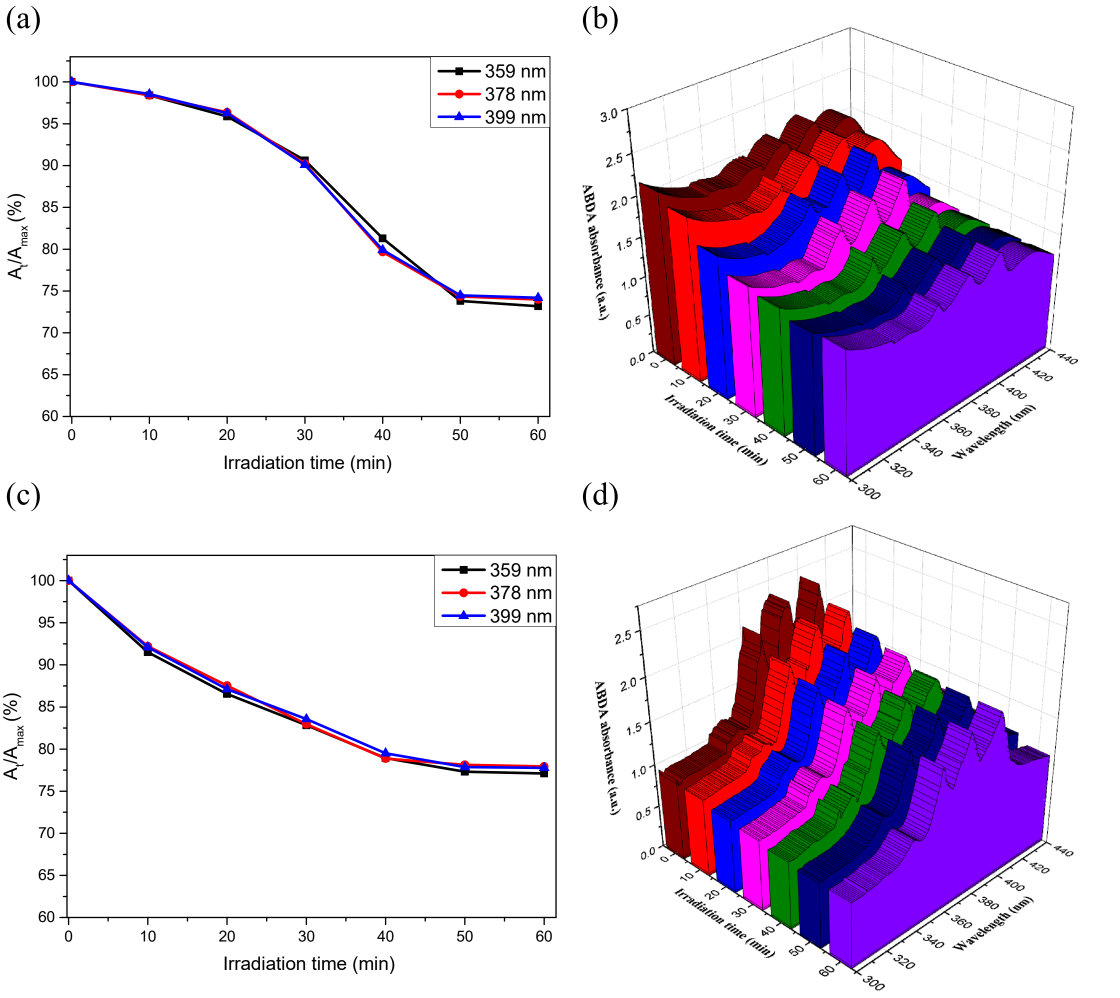

Figure S6. Under NIR exposure, the UV-vis absorbance change of Ho^{3+}-doped UCNP@mSiO2/RB donor-acceptor system (a) as a function of irradiation time and (b) as a function of irradiation time and absorption wavelength. The absorbance change of Er^{3+}-doped UCNP@mSiO2/Ce6 donor-acceptor system (c) as a function of irradiation time and (d) as a function of irradiation time and absorption wavelength.

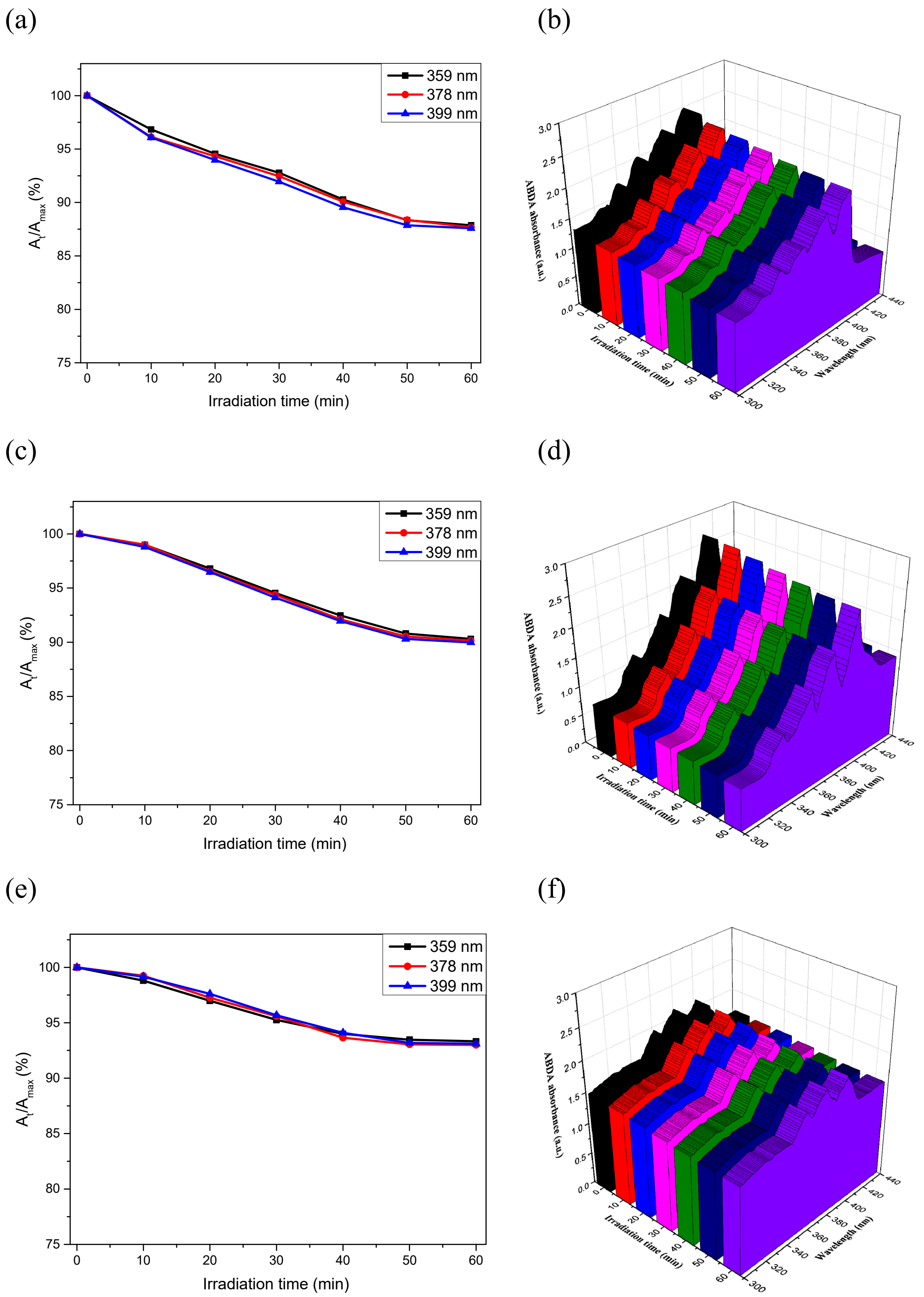

Figure S7. Under NIR exposure, the UV-vis absorbance change of oleate-capped Ho^{3+}-doped UCNP/MC 540 donor-acceptor system (a) as a function of irradiation time and (b) as a function of irradiation time and absorption wavelength. The absorbance change of oleate-capped Ho^{3+}-doped UCNP/RB donor-acceptor system (c) as a function of irradiation time and (d) as a function of irradiation time and absorption wavelength. The absorbance change of oleate-capped Er^{3+}-doped UCNP/Ce6 donor-acceptor system (e) as a function of irradiation time and (f) as a function of irradiation time and absorption wavelength.

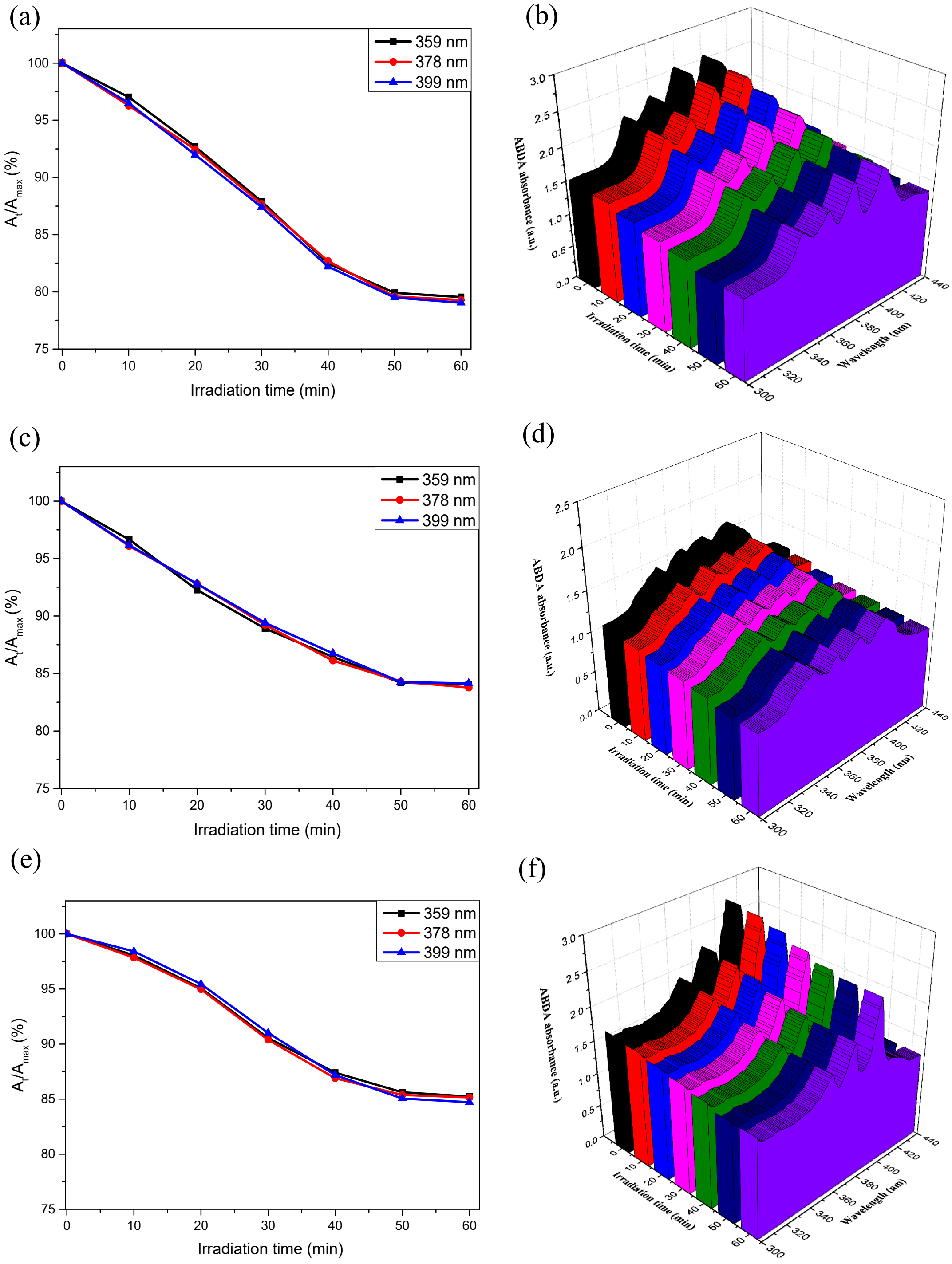

Figure S8. Under NIR exposure, the UV-vis absorbance change of ligand-free Ho^{3+}-doped UCNP/MC 540 donor-acceptor system (a) as a function of irradiation time and (b) as a function of irradiation time and absorption wavelength. The absorbance change of ligand-free Ho^{3+}-doped UCNP/RB donor-acceptor system (c) as a function of irradiation time and (d) as a function of irradiation time and absorption wavelength. The absorbance change of ligand-free Er^{3+}-doped UCNP/Ce6 donor-acceptor system (c) as a function of irradiation time and (d) as a function of irradiation time and absorption wavelength.
